# Spatial Clustering and Risk Factors for Malaria Infections and Marker of Recent Exposure to *Plasmodium falciparum* from a Household Survey in Artibonite, Haiti

**DOI:** 10.4269/ajtmh.22-0599

**Published:** 2023-06-05

**Authors:** Karen E. S. Hamre, Amber M. Dismer, Eric Rogier, Lotus L. van den Hoogen, John Williamson, Nishant Kishore, Anyess Travers, Kathleen McGee, Baby Pierre, Bernadette Fouché, Daniel Impoinvil, Kathleen Holmes, Gillian Stresman, Thomas Druetz, Thomas P. Eisele, Chris Drakeley, Jean Frantz Lemoine, Michelle A. Chang

**Affiliations:** ^1^ Malaria Branch, Division of Parasitic Diseases and Malaria, Center for Global Health, Centers for Disease Control and Prevention, Atlanta, Georgia;; ^2^ CDC Foundation, Atlanta, Georgia;; ^3^ Emergency Response and Recovery Branch, Division of Global Health Protection, Center for Global Health, Centers for Disease Control and Prevention, Atlanta, Georgia;; ^4^ London School of Hygiene & Tropical Medicine, London, United Kingdom;; ^5^ Center for Applied Malaria Research and Evaluation, Tulane University School of Public Health and Tropical Medicine, New Orleans, Louisiana;; ^6^ Population Services International/Organisation Haïtienne de Marketing Social pour la Santé, Peguy-ville, Haiti;; ^7^ Programme National de Contrôle de la Malaria, Ministère de la Santé Publique et de la Population, Port-au-Prince, Haiti;; ^8^ University of Montreal School of Public Health, Montreal, Canada

## Abstract

Targeting malaria interventions in elimination settings where transmission is heterogeneous is essential to ensure the efficient use of resources. Identifying the most important risk factors among persons experiencing a range of exposure can facilitate such targeting. A cross-sectional household survey was conducted in Artibonite, Haiti, to identify and characterize spatial clustering of malaria infections. Household members (*N* = 21,813) from 6,962 households were surveyed and tested for malaria. An infection was defined as testing positive for *Plasmodium falciparum* by either a conventional or novel highly sensitive rapid diagnostic test. Seropositivity to the early transcribed membrane protein 5 antigen 1 represented recent exposure to *P. falciparum*. Clusters were identified using SaTScan. Associations among individual, household, and environmental risk factors for malaria, recent exposure, and living in spatial clusters of these outcomes were evaluated. Malaria infection was detected in 161 individuals (median age: 15 years). Weighted malaria prevalence was low (0.56%; 95% CI: 0.45–0.70%). Serological evidence of recent exposure was detected in 1,134 individuals. Bed net use, household wealth, and elevation were protective, whereas being febrile, over age 5 years, and living in either households with rudimentary wall material or farther from the road increased the odds of malaria. Two predominant overlapping spatial clusters of infection and recent exposure were identified. Individual, household, and environmental risk factors are associated with the odds of individual risk and recent exposure in Artibonite; spatial clusters are primarily associated with household-level risk factors. Findings from serology testing can further strengthen the targeting of interventions.

## INTRODUCTION

Hispaniola, composed of Haiti and the Dominican Republic, remains the last malaria-endemic island in the Caribbean; both countries are committed to eliminating malaria.^1^^,^^2^ In 2017, over 99% of reported malaria cases in Hispaniola were due to *Plasmodium falciparum*, and 98% of confirmed cases were from Haiti (*N* = 18,843).^3^ In Haiti, *Anopheles albimanus* mosquitoes, which have zoophilic and exophilic tendencies, are the main malaria vector.^4^^–^^6^ Generally, the niche of *An. albimanus* is considered to be in low-lying areas with upper limits of 500 m from sea level in Haiti; however, higher elevations of up to 762 m from sea level have also been reported on the island.^4^^,^^7^ In general, malaria transmission is bimodal, after the rainy seasons in November to January and May to July.^8^ Overall, malaria transmission is low, with recent national estimates from population-based surveys in 2011, 2012, and 2015 reporting a malaria parasite prevalence of 0.4–0.5% by polymerase chain reaction assays.^1^^,^^9^^–^^11^ Still, malaria transmission across Haiti is heterogeneous and focal, with reported estimates in some populations in Artibonite of 2.8 and 3.1%, ranging up to 40.7 and 46.3% in Grand’Anse.^12^^–^^14^

From 2015 to 2020, Malaria Zero Consortium partners worked to accelerate malaria elimination in Haiti, which requires reducing the number of undetected infections as part of the strategy.^15^ Haiti’s National Malaria Control Program (French: Program National de Contrôle de la Malaria; PNCM) National Strategic Plan for the Elimination of Malaria in Haiti 2016–2022 aligns with the World Health Organization strategic framework for malaria elimination.^15^^,^^16^ The plan includes an overarching strategy to aggressively identify and eliminate parasite infections in the human population, and parasite reservoirs in the vector population, via priority interventions in selected communities; doing so would rapidly clear all stages of the parasite and prevent onward transmission. These priority interventions include enhanced surveillance and case management, targeted vector control measures (i.e., distribution of long-lasting insecticidal nets; LLINs) and larval source management, and targeted mass drug administration (MDA) campaigns.^17^ Strategies for delivering interventions in low-transmission settings must be increasingly targeted toward smaller areas with increased malaria risk and/or toward more specific populations.^18^ The primary objective of this survey was to identify geospatial characteristics of high-transmission foci and the risk factors of the associated populations to target future interventions.

## MATERIALS AND METHODS

### Survey setting.

**Figure 1.**
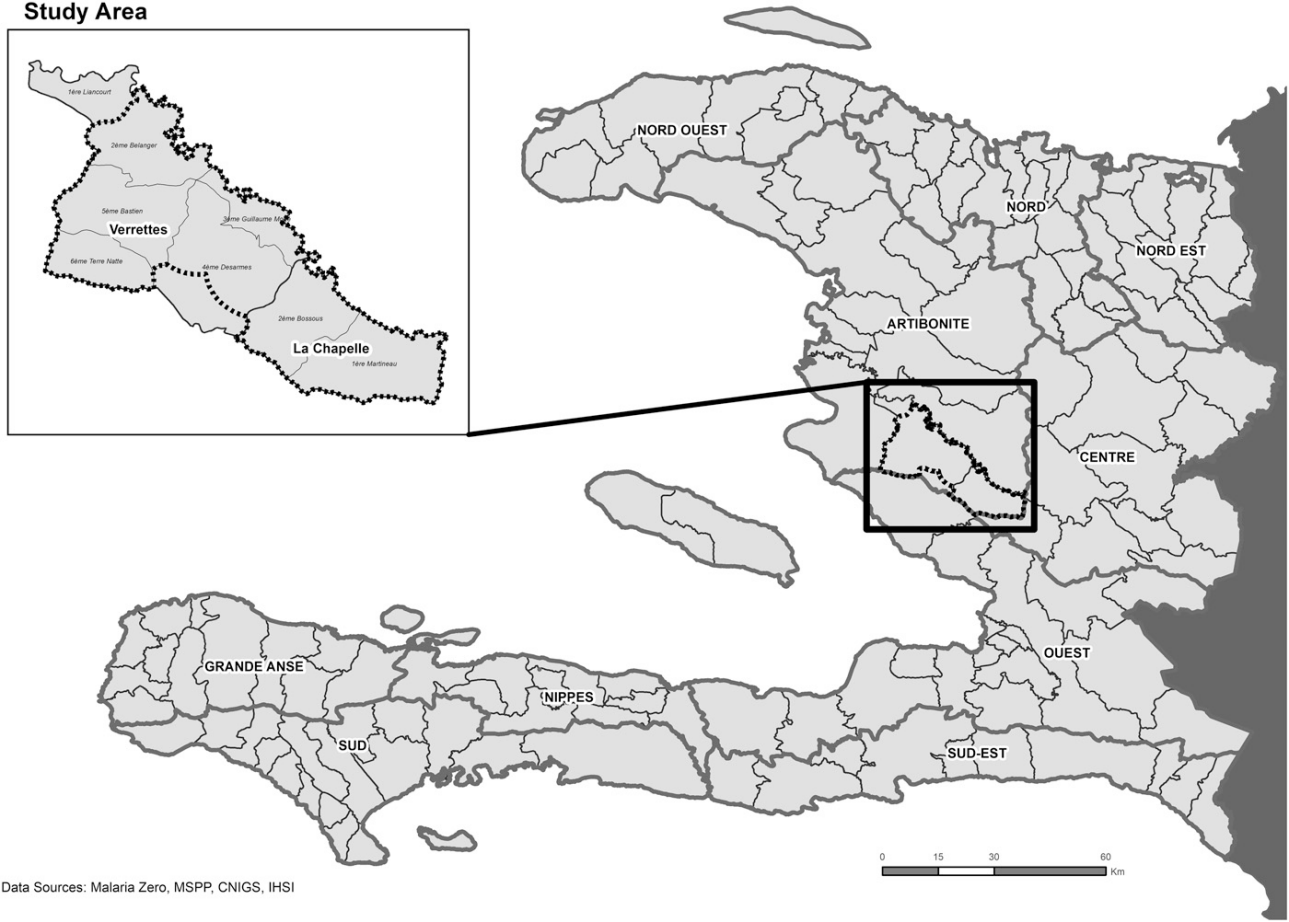
Study area boundaries in Verrettes and La Chapelle, Artibonite, Haiti.

An observational cross-sectional household survey to identify and characterize spatial clustering of malaria infections, and populations most at risk for malaria, was conducted in the communes of La Chapelle and Verrettes in Artibonite Department, Haiti (Figure 1). The study area is land locked, with the Artibonite River flowing through it; there are valleys and mountainous terrain (elevation ranges from sea level to ∼1,430 m). In collaboration with the PNCM, this study area was selected for its accessibility and relatively high number of malaria cases in the Haitian context. The 2016 incidence of malaria in these communes was 3.9 and 2.1 per 1,000 persons, respectively, whereas the overall malaria incidence across Haiti was 2 per 1,000 persons (French: Ministère de la Santé Publique et de la Population; MSPP, unpublished data). Other potential study areas with higher reported annual incidence (e.g., in Grand’Anse, Nippes, and Sud departments) were excluded from consideration for feasibility reasons after Hurricane Matthew made landfall in October 2016 and caused substantial damage and displaced populations.

### Survey design and data collection.

A complete georeferenced census of the study area was conducted from June 1 to 22, 2017, prior to the household survey. The study area was subdivided into 1 × 1-km^2^ contiguous operational units (OUs); 476 OUs had inhabited, residential households ranging from 1 to 1,627 households. Households were the primary sampling unit and were selected by simple random sampling within each OU to ensure spatial distribution across the study area. To maximize the precision of estimates, sample sizes for each OU were dependent on the population size of the OU, which were categorized into three strata: 1) large OUs (> 200 households); 2) medium OUs (20–200 households); and 3) small OUs (< 20 households). Ten percent of households in large OUs were sampled, 20 households (10–100%) were sampled in medium OUs, and 100% of households in small OUs were sampled. An additional 5% of households in large and medium OUs were oversampled to accommodate expected household-level refusals.

Sampled households were visited from July 17 to October 4, 2017 (no visits occurred from September 6 to 24, 2017, because of hurricane threats). Maps of the study area with houses, roads, and key landmarks were created and uploaded onto tablets to assist in navigation to the sampled households using the PDF Maps application (Avenza Systems, Toronto, Ontario, Canada). The survey instrument and sample tracking were programmed into CommCare (Dimagi). Data collected during the census, including household coordinates, head of household name, and household description, were uploaded into CommCare to assist the teams in locating the selected households. An interviewer and a laboratory technician visited the sampled households and obtained consent, administered the questionnaire, tested for malaria using rapid diagnostic tests (RDTs), and collected blood samples for serology testing.

The survey consisted of two sets of questions, one for the head of household (HoH) and one for the primary caretaker (CT), if different. The HoH and/or CT were asked questions about malaria knowledge; information, education, and communication; care-seeking behavior; mobility; acceptability of an MDA; and socioeconomic status. Additionally, the demographics of each household member, along with bed net use (last night), recent travel (outside the home subcommune within 3 months), and previous residence (within 24 months) were collected. All consenting household members had their axillary temperature measured and a finger prick blood sample for malaria diagnosis taken by a conventional RDT (cRDT; SD BIOLINE Malaria Ag P.f, 05FK50; Standard Diagnostics Suwon City, Republic of Korea) and a highly sensitive RDT (HS-RDT; Alere Malaria Ag P.f, 05FK141; Standard Diagnostics).^19^ An individual with axillary temperature ≥ 38.0°C was defined as febrile. Household members who tested positive for malaria by cRDT were provided treatment per Ministry of Health guidelines (HS-RDTs were conducted for research purposes only). Up to 300 µL of blood was collected in an ethylenediaminetetraacetate capillary tube for filter paper (FP) spotting at a central laboratory facility. Nightly, five 50-µL aliquots of blood from each sample were pipetted onto a Whatman 903 FP card (GE Healthcare, Chicago, IL), dried at ambient temperature, and individually packaged with desiccant the next day. Dried blood spots were stored at ambient temperature protected from light and transported weekly to the National Public Health Laboratory (French: Laboratoire National de Santé Publique; LNSP) in Port-au-Prince.

At the LNSP, antibody responses (immunoglobulin G; IgG) to early transcribed membrane protein 5 antigen 1 (Etramp 5 ag 1) and *Schistosoma japonicum* glutathione *S*-transferase (GST) were measured using a multiplex bead assay with GST as a nonbinding control because Etramp 5 ag 1 is expressed with a GST purification tag. Assay protocol and quality control processes were previously described.^20^^,^^21^ Median fluorescence intensity (MFI) was recorded using MAGPIX with Bio-Plex Manager MP software (Bio-Rad, Hercules, CA).

Up to three visits per household were attempted before the household was recorded as unavailable. Similarly, up to two return visits for absent household members who were expected to return within 2 days of the initial survey were attempted following the same procedures described above.

### Study ethical review.

For survey participation and malaria diagnostic testing, informed consent from HoHs and/or CTs for and their children < 18 years of age, consent from all individual adult members, and assent from children aged 7–17 years were obtained and documented electronically. The study was approved by the Haitian Ministry of Public Health and Population Bioethics Committee, the Institutional Review Board of the CDC, and the London School of Hygiene & Tropical Medicine Ethics Committee.

### Statistical and spatial analysis.

A malaria infection was defined as an individual testing positive for *P. falciparum* by either cRDT or HS-RDT. Although the national policy in Haiti only allowed the use of cRDTs for the diagnosis and treatment of malaria, a nested study comparing the performance of cRDT versus HS-RDT provided results from the novel HS-RDTs, which have been shown to detect slightly more infections.^19^ Seropositivity to Etramp 5 ag 1 was selected to represent recent exposure to *P. falciparum* (i.e., aiming to represent past infection in the prior year).^22^^,^^23^ Antibodies to Etramp 5 ag 1 were shown to correlate with individual malaria incidence in the prior year in Ugandan children and population-level seasonal incidence in all ages in The Gambia.^22^^,^^24^ Etramp 5 ag 1 antibodies were also associated with seasonal changes in malaria transmission at the village level in The Gambia.^25^ A finite mixture model was used to identify two components in the log_10_-transformed MFI participant data, and the threshold for seropositivity was defined as the mean of the lower distribution plus 5 SDs.^23^ For serological analysis, samples from 73 individuals with high responses to GST (i.e., evidence for nonspecific IgG binding) and 596 individuals < 1 year of age (because of the possibility of maternally derived antibodies to malaria) were excluded.^21^ Clusters of malaria infections and recent exposure to *P. falciparum* were identified using purely retrospective spatial models with Poisson distributions in SaTScan (Martin Kulldorff with Information Management Services Inc., Boston, MA). Unweighted estimates of parasite prevalence by OU were calculated and mapped. The overall weighted parasite prevalence was estimated using the survey estimation commands in Stata SE version 15.1 (StataCorp, College Station, TX) to account for the complex survey design (i.e., sampling weights and clustering at the household level).

The univariate and adjusted associations between infections and concordant negatives (those who tested negative by cRDT and HS-RDT) among potential individual, household, and environmental risk factors for individual malaria risk, and for risk of living in a spatial infection cluster, were evaluated using the survey estimation commands in Stata SE version 15.1. For adjusted models, sex and age category were selected a priori. These methods were repeated for the serologic marker of recent exposure analyses.

In addition to demographic and individual characteristics, several covariates were tested in the models. Euclidian distances from the household to each of the nearest health facility, river, and road were derived using ArcGIS Desktop version 10.5.1 (ESRI, Redlands, CA). Population density by OU was determined using census data. A household wealth index categorized into quintiles was derived using principal-components analysis of several fields that took into account ownership of durable assets (e.g., radio, mobile phone, bicycle, motorcycle, or scooter) and access to utilities and infrastructure (e.g., electricity, source of water, and sanitation facility) as per the approach used during the Demographic Health Survey.^26^ Housing characteristics, such as roof, wall, and floor material, and ownership of livestock or agricultural land were independently evaluated, each of which may be independently associated with the risk of malaria. HoH occupation and the education levels of the HoH and CT were only asked in the respective survey; hence, these fields were only included in the univariate analyses.

Weather and environmental variable data used in this analysis were extracted from the Climate Hazard Group Infrared Precipitation (CHIRPS) and Moderate Resolution Imaging Spectroradiometer (MODIS) datasets. CHIRPS data have been made available by the US Geological Survey and involve satellite data merged with archived station data.^27^ Specifically, by using GPS coordinates for each household, monthly mean rainfall data from June to October 2017 were extracted from CHIRPS at 5-km resolution. Monthly means (prior month and same month) of each of the enhanced vegetation index, tasseled cap wetness index (a measure of soil and canopy moisture), tasseled cap brightness (a measure of soil brightness), and day, night, and diurnal difference (a proxy for moisture) land surface temperature data from June to October 2017 were derived at 1-km resolution from MODIS National Aeronautics and Space Administration satellite data and extracted for each household.^28^

The method of purposeful selection of covariates was used to determine the final adjusted models.^29^ Statistical analyses were performed using Stata SE version 15.1; *P* < 0.05 was considered statistically significant.

## RESULTS

### Households.

Of the 33,060 inhabited households identified during the 2017 complete census in the study area, 8,818 were sampled (1,448 from 31 large OUs, 5,937 from 283 medium OUs, and 1,433 from 162 small OUs) to participate in the household survey. Among these sampled households, 7,338 (83.2% overall, 1,151 [79.5%] from large OUs, 5,020 [84.6%] from medium OUs, and 1,167 [81.4%] from small OUs) had an HoH and/or CT available to approach to consent to participate; 1,465 (16.6%) were unavailable. Survey data were lost for 15 (0.2%) households because of application or tablet malfunctions. The reasons for a household’s unavailability are categorized in Supplemental Table 1, with travel being the most common reason documented (55.1%). Fifteen percent of selected households were reported as abandoned or locked since the time of the census.

Supplemental Figure 1 depicts a flow diagram from the number of sampled households to the numbers of completed surveys. In summary, completed surveys were recorded from 6,962 households in total, consisting of 5,952 HoH surveys and 2,537 CT surveys. The average household size was 3.8 members (SD = 2.1).

During the initial visit, 6,020 (68.3%) households were closed, meaning they were available to provide consent or refuse to participate in the survey. A second visit was required to close 961 (10.9%) households, and 1,822 (20.7%) households required three visits to close.

### Individuals.

Among the 6,962 households with completed surveys, 26,351 household members (present and absent) were rostered and 21,813 (82.8%) were present and consented to a blood draw (Supplemental Figure 2). More males (2,419; 65.3%) than females (1,288; 34.7%) were absent (*P <* 0.001). Nearly one-third (1,190; 32.1%) of absent individuals were traveling overnight outside his/her home subcommune, and 36.1% of these individuals were traveling outside of Artibonite Department. Several individuals refused a blood draw because they were recently tested for malaria (226; 27.2%). A comprehensive list of reasons for refusal is categorized in Supplemental Table 2.

The median age of household members was 19 years (interquartile range [IQR]: 8–40) and 53.9% were female. Only 95 (0.4%) were febrile by axillary temperature, and 5.9% reported sleeping under a bed net the night before the survey. The vast majority (88.3%) reported living in the same house location during the past 24 months, and recent travel within the past 3 months was low (3.7%). Characteristics of consenting individuals are presented in Table 1.

**Table 1:** Demographics and characteristics of consenting household members

Characteristics	*N* = 21,813 *n* (%) or median (IQR)
Sex, female	11,746 (53.9)
Age (years)	19 (8–40)
Age category (years)	
< 5	3,463 (15.9)
5 to < 15	5,676 (26.0)
15+	12,671 (58.1)
Do not know	3 (0.01)
Febrile, axillary temperature ≥ 38.0°C	95 (0.4)
Did you sleep under a bed net last night? Yes	1,296 (5.9)
Live in same household during past 24 months?* Yes	19,268 (88.3)
Recent travel within last 3 months? Yes	810 (3.7)

IQR = interquartile range.

* If a child was < 24 months old, a “yes” indicated the child lived in the same household during their lifetime.

### Blood samples, testing, and spatial clusters.

A total of 21,830 household members had RDT results captured, and 21,801 had a blood sample spotted onto FP for serology testing. Supplemental Figure 2 illustrates the flow diagram of rostered individuals and samples collected for testing. Data from 21,771 individuals with household GPS coordinates and survey data are included in the risk factor for malaria analysis. Among these, 21,102 (96.8% of samples) were individuals ≥ 1 year old who had acceptable negative responses to the GST internal assay control and linked to households with survey data for use in the analysis of the risk factor for marker of recent exposure to *P. falciparum*.^21^

**Figure 2.**
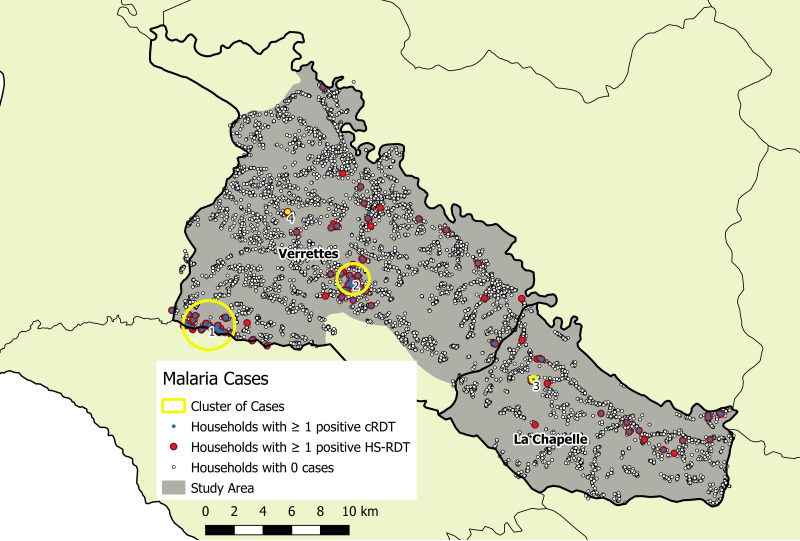
Spatial clusters of RDT positives, La Chapelle and Verrettes, Artibonite, Haiti. cRDT = conventional rapid diagnostic test; HS-RDT = highly sensitive rapid diagnostic test.

**Figure 3.**
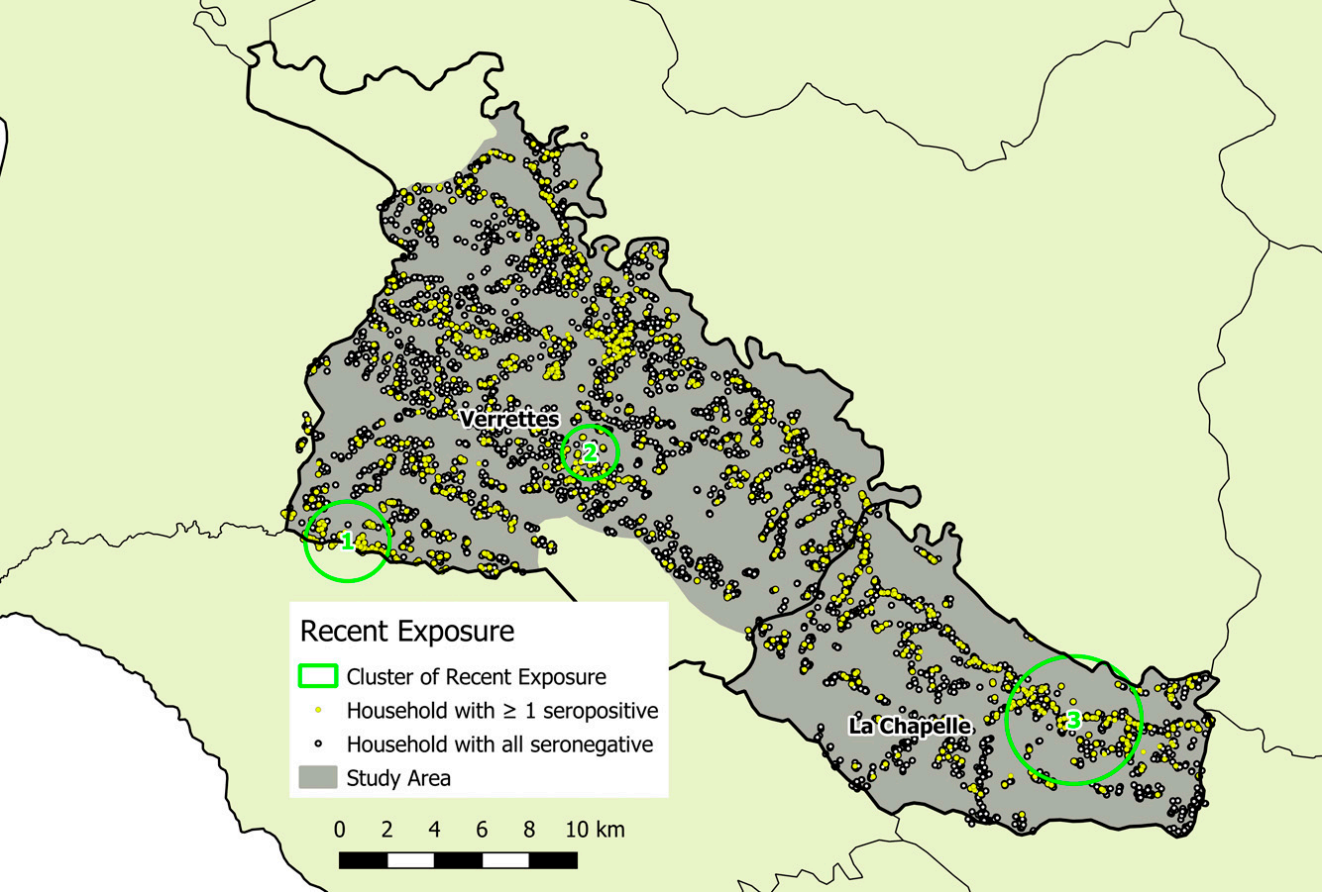
Spatial clusters of seropositives for IgG indicating recent exposure to *Plasmodium falciparum*, La Chapelle and Verrettes, Artibonite, Haiti.

**Figure 4.**
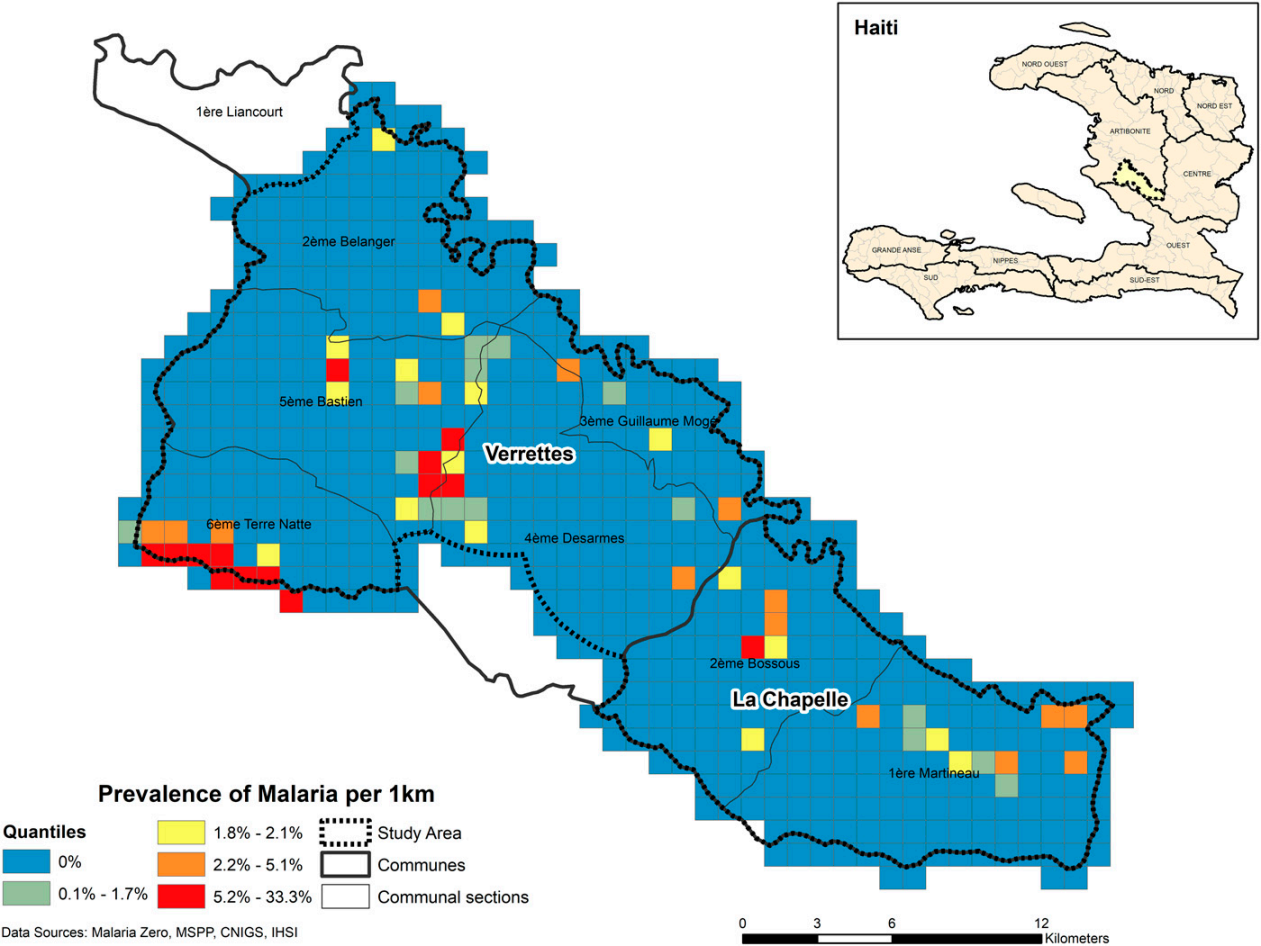
Unweighted prevalence of malaria, by operational unit (OU) 1x1km grid, Artibonite, Haiti.

Overall, there were *161 P. falciparum* infections identified (125 by cRDT and 160 by HS-RDT; 124 were concordant positives). The median age of infected individuals was 15 years (range: 0.75–81; IQR: 7–30). Weighted malaria prevalence in the study area was low (0.56%; 95% CI: 0.45–0.70%), yet four significant, including two predominant, spatial infection clusters were identified in Verrettes (Figure 2, Table 2). Among all detected infections, 65.8% (*N* = 106) were within identified spatial infection clusters. Recent exposure, defined as Etramp 5 ag 1 seropositivity, was detected in 1,134 individuals (5.2%). Three significant spatial clusters of marker of recent exposure to *P. falciparum* were identified: two in similar locations as the predominant spatial infection clusters in Verrettes, plus one in La Chapelle (Figure 3, Table 2). Unweighted parasite prevalence estimates by OU are presented in Figure 4.

**Table 2:** Spatial case clusters detected by RDT* or SERO marker of recent exposure to *P. falciparum*

Cluster	Radius (km)	Population	No. of cases	No. of expected cases	Relative risk	*P* value
RDT1	1.71	478	64	3.53	29.39	< 0.001
RDT2	1.07	267	31	1.97	19.21	< 0.001
RDT3	0.20	23	7	0.17	42.98	< 0.001
RDT4	0.07	14	4	0.10	39.59	0.043
SERO1	1.68	411	144	22.09	7.32	< 0.001
SERO2	1.11	193	46	10.37	4.58	< 0.001
SERO3	2.69	972	91	52.23	1.81	0.018

RDT = rapid diagnostic test; SERO = serology.

* Positive by either conventional RDT or highly sensitive RDT.

### Risk factor analyses.

In the final adjusted model, the protective effect of bed net use (adjusted odds ratio [aOR]: 0.28; 95% CI: 0.09–0.92; *P* = 0.04) and wealth (e.g., aOR fourth quintile versus poorest quintile: 0.28; 95% CI: 0.09–0.85; *P* = 0.03 and aOR wealthiest quintile versus poorest quintile: 0.37; 95% CI: 0.13–1.02; *P* = 0.06) were maintained (Table 3). Also, being febrile was significantly associated with malaria infection (aOR: 3.71; 95% CI: 1.33–10.4; *P* = 0.01), whereas elevation was protective (aOR per 100 m: 0.73; 95% CI: 0.66–0.81; *P* ≤ 0.001). Individuals living in a household with rudimentary wall material had an increased odds of malaria as compared with those living in a household with finished wall material (i.e., cement, stone, or tile) (aOR: 1.90; 95% CI: 1.11–3.23; *P* = 0.02). Living in a household farther from the nearest road increased the odds of malaria (aOR per 100 m: 1.05; 95% CI: 1.03–1.07; *P* < 0.001). Significant climate variables included in the final model were prior month mean rainfall (aOR: 0.78; 95% CI: 0.73–0.84; *P* < 0.001) and enhanced vegetation index during the same month as the RDT (aOR: 0.79; 95% CI: 0.66–0.95; *P* = 0.01). In addition, as compared with individuals aged < 5 years, individuals aged 5 to < 15 years and individuals aged 15+ had increased odds of malaria (aOR: 1.99; 95% CI: 1.16–3.43; *P* = 0.01 and aOR: 1.79; 95% CI: 1.04–3.09; *P* = 0.04, respectively).

**Table 3:** Risk factors for malaria infection status in the study area, Artibonite, Haiti, *N* = 21,771*

Risk factor	Case (cRDT- or HS-RDT-positive) survey estimate, % or mean (95% CI) *N* = 161*	Concordant RDT-negative survey estimate, % or mean (95% CI) *N* = 21,610*	Univariate *P* value	Adjusted OR (95% CI)	Adjusted *P* value
Individual level					
Sex, male	54.0 (43.9–63.8)	45.1 (44.3–45.9)	0.08	1.39 (0.90–2.13)	0.13
Age category (years)			Overall 0.09		
0 to < 5	10.7 (6.8–16.4)	14.8 (14.2–15.4)	Ref	Ref	Ref
5 to < 15	32.1 (23.7–41.8)	24.8 (24.1–25.5)	0.03	1.99 (1.16–3.43)	0.01
15+	57.2 (47.2–66.7)	60.5 (59.7–61.3)	0.31	1.79 (1.04–3.09)	0.04
Febrile, yes	1.8 (0.6–5.2)	0.34 (0.26–0.45)	0.14	3.71 (1.33–10.4)	0.01
Did you sleep under a bed net last night? Yes	1.9 (0.6–5.7)	8.8 (8.0–9.7)	< 0.001	0.28 (0.09–0.92)	0.04
Live in same household during past 24 months? Yes	86.7 (77.0–92.7)	85.9 (84.9–86.9)	0.83	–	–
Recent travel within last 3 months? Yes	2.4 (0.8–7.4)	4.7 (4.3–5.1)	0.12	–	–
Household level					
Occupation of head of household (*N* = 18,579), agricultural/farmer	67.8 (56.5–77.3)	51.0 (49.1–52.9)	0.007	–	–
Head of household education level (*N* = 18,563), never attended	49.7 (37.8–61.7)	48.7 (46.8–50.5)	0.86	–	–
Caretaker education level (*N* = 9,890), never attended	53.4 (37.1–69.1)	49.9 (47.1–52.7)	0.20	–	–
Wealth index (quintiles)			Overall < 0.001		
Poorest	48.5 (39.0–58.0)	19.8 (18.8–20.9)	Ref	Ref	Ref
2nd	20.4 (12.9–30.6)	20.0 (18.8–21.3)	0.002	0.65 (0.38–1.13)	0.13
Middle	16.6 (9.9–26.6)	20.0 (18.6–21.6)	< 0.001	0.60 (0.30–1.20)	0.15
4th	6.5 (2.4–16.4)	20.0 (18.6–21.6)	< 0.001	0.28 (0.09–0.85)	0.03
Wealthiest	8.0 (3.4–18.0)	20.1 (18.5–21.7)	< 0.001	0.37 (0.13–1.02)	0.06
Livestock ownership? Yes	73.7 (62.8–82.3)	69.3 (67.7–70.9)	0.38	–	–
Agricultural land ownership? Yes	68.6 (57.1–78.2)	68.3 (66.6–69.9)	0.96	–	–
Roof material					
Thatched, sod, mat, wood	9.0 (4.8–16.4)	2.8 (2.4–3.3)	0.03	–	–
Metal, cement, shingles	91.0 (83.6–95.2)	97.2 (96.7–97.6)			
Wall material					
None, bamboo, earth, mud, wood	67.4 (57.2–76.2)	40.3 (38.7–41.9)	< 0.001	1.90 (1.11–3.23)	0.02
Cement, stone, tile	32.6 (23.8–42.8)	59.7 (58.1–61.3)			
Floor material					
Earth, sand, wood	84.2 (73.2–91.2)	62.2 (60.5–63.9)	< 0.001	–	–
Cement, stone, tile	15.8 (8.8–26.8)	37.8 (36.1–39.5)			
Elevation (m)	365.3 (327.5–403.0)	274.8 (266.2–283.4)	< 0.001	0.73 (0.66–0.81)†	< 0.001
Distance to nearest health facility (m)	3,503.5 (3207.4–3799.7)	2,577.3 (2524.8–2629.8)	< 0.001	–	–
Distance to nearest water source (m)	869.2 (669.1–1069.3)	826.1 (803.7–848.6)	0.675	–	–
Distance to nearest road (m)	1,257.4 (1048.0–1466.9)	480.0 (457.5–502.4)	< 0.001	1.05 (1.03–1.07)†	< 0.001
Population density	705.5 (475.0–936.0)	1,019.4 (974.1–1064.7)	0.05	–	–
Environmental					
Monthly mean rainfall (mm), prior month	177.3 (169.9–184.7)	204.5 (203.7–205.3)	< 0.001	0.78 (0.73–0.84)‡	< 0.001
Monthly mean rainfall (mm), same month	184.6 (177.6–191.5)	202.4 (201.7–203.1)	< 0.001	–	–
Enhanced vegetation index, prior month	0.48 (0.46–0.49)	0.48 (0.48–0.48)	0.29	–	–
Enhanced vegetation index, same month	0.49 (0.48–0.50)	0.49 (0.49–0.50)	0.32	0.79 (0.66–0.95)§	0.01
Tasseled cap wetness index, prior month	−0.25 (−0.26–−0.25)	−0.25 (−0.25–−0.25)	0.12	–	–
Tasseled cap wetness index, same month	−0.24 (−0.24–−0.23)	−0.23 (−0.23–−0.23)	< 0.001	–	–
Tasseled cap brightness index, prior month	0.57 (0.57–0.58)	0.57 (0.57–0.57)	0.17	–	–
Tasseled cap brightness index, same month	0.56 (0.56–0.57)	0.55 (0.55–0.55)	0.002	–	–
Day land surface temperature (°C), prior month	30.2 (29.9–30.5)	30.9 (30.8–30.9)	< 0.001	–	–
Day land surface temperature (°C), same month	29.5 (29.3–29.8)	30.2 (30.2–30.3)	< 0.001	–	–
Night land surface temperature (°C), prior month	20.2 (20.0–20.5)	21.0 (20.9–21.0)	< 0.001	–	–
Night land surface temperature (°C), same month	21.1 (20.9–21.3)	21.6 (21.6–21.7)	< 0.001	–	–
Diurnal difference land surface temperature (proxy for moisture), prior month	9.9 (9.7–10.2)	9.9 (9.9–10.0)	0.89	–	–
Diurnal difference land surface temperature (proxy for moisture), same month	8.5 (8.3–8.6)	8.6 (8.6–8.6)	0.15	–	–

cRDT = conventional rapid diagnostic test; HS-RDT = highly sensitive rapid diagnostic test; OR, odds ratio; Ref = reference.

* Unweighted sample sizes.

† Per 100 m.

‡ Per centimeter.

§ Standardized to mean 0 and SD 1.

In the final adjusted model evaluating risk factors for recent exposure, wealth index, elevation, distance to nearest road, and monthly mean rainfall (prior month) were similarly included. Evidence for a protective effect of wealth was weaker in the recent exposure analysis, although as compared with those living in households among the poorest quintile there was a significant protective effect among individuals living in households in the median quintile (aOR: 0.77; 95% CI: 0.60–0.99; *P* = 0.04). A less pronounced but still significant protective effect of elevation on recent exposure was also seen (aOR per 100 m: 0.90; 95% CI: 0.87–0.94; *P* < 0.001). Living farther from the nearest road increased the odds of recent exposure (aOR per 100 m: 1.03; 95% CI: 1.02–1.04; *P* < 0.001). Finally, as compared with individuals aged < 5 years, individuals aged 15+ had increased odds of malaria (aOR: 2.44; 95% CI: 1.88–3.17; *P* < 0.001) (Table 4).

**Table 4:** Risk factors for the presence of marker of recent exposure to P. falciparum* in the study area, Artibonite, Haiti, *N* = 21,102†

Risk factor	Recent exposure (yes) survey estimate, % or mean (95% CI) *N* = 1,134†	Recent exposure (no) survey estimate, % or mean (95% CI) *N* = 19,968†	Univariate *P* value	Adjusted OR (95% CI)	Adjusted *P* value
Individual level					
Sex, male	44.9 (41.2–48.6)	45.1 (44.3–46.0)	0.90	1.06 (0.91–1.24)	0.47
Age category (years)			Overall < 0.001		
1 to < 5	8.1 (6.4–10.2)	15.0 (14.4–15.7)	Ref	Ref	Ref
5 to < 15	16.8 (14.3–19.5)	25.3 (24.6–26.1)	0.17	1.24 (0.92–1.68)	0.15
15+	75.1 (72.0–78.0)	59.7 (58.8–60.5)	< 0.001	2.44 (1.88–3.17)	< 0.001
Febrile, yes	0.58 (0.23–1.47)	0.33 (0.25–0.44)	0.36	–	–
Did you sleep under a bed net last night? Yes	8.9 (6.8–11.5)	8.7 (7.9–9.6)	0.92	–	–
Live in same household during past 24 months? Yes	88.1 (85.3–90.4)	85.8 (84.7–86.8)	0.07	–	–
Recent travel within last 3 months? Yes	4.9 (3.5–6.8)	4.7 (4.3–5.2)	0.86	–	–
Household level					
Occupation of head of household (*N* = 18,579), agricultural/farmer	53.4 (49.3–57.6)	50.7 (48.8–52.6)	0.20	–	–
Head of household education level (*N* = 18,563), never attended	50.3 (46.2–54.4)	48.4 (46.5–50.3)	0.37	–	–
Caretaker education level (*N* = 9,890), never attended	53.5 (47.6–59.3)	49.8 (47.0–52.6)	0.23	–	–
Wealth index (quintiles)			Overall 0.03		
Poorest	24.1 (21.5–27.0)	19.6 (18.6–20.7)	Ref	Ref	Ref
2nd	19.6 (16.9–22.5)	20.0 (18.8–21.3)	0.03	0.85 (0.69–1.06)	0.15
Middle	17.3 (14.3–20.7)	20.1 (18.6–21.6)	0.003	0.77 (0.60–0.99)	0.04
4th	19.0 (16.0–22.4)	20.1 (18.6–21.7)	0.02	0.84 (0.65–1.07)	0.16
Wealthiest	20.1 (16.8–23.8)	20.2 (18.6–21.9)	0.08	0.87 (0.66–1.14)	0.32
Livestock ownership? Yes	69.0 (65.2–72.6)	69.4 (67.7–71.0)	0.86	–	–
Agricultural land ownership? Yes	70.4 (66.5–73.9)	68.2 (66.5–69.9)	0.24	–	–
Roof material					
Thatched, sod, mat, wood	3.6 (2.5–5.3)	2.7 (2.3–3.3)	0.20	–	–
Metal, cement, shingles	96.4 (94.7–97.5)	97.3 (96.8–97.7)			
Wall material					
None, bamboo, earth, mud, wood	43.1 (39.6–46.7)	40.2 (38.6–41.8)	0.13	–	–
Cement, stone, tile	56.9 (53.3–60.4)	59.8 (58.2–61.4)			
Floor material					
Earth, sand, wood	65.7 (61.9–69.3)	61.9 (60.1–63.6)	0.06	–	–
Cement, stone, tile	34.3 (30.7–38.1)	38.1 (36.4–39.9)			
Elevation (m)	281.9 (263.1–300.8)	274.6 (265.8–283.3)	0.50	0.90 (0.87–0.94)‡	< 0.001
Distance to nearest health facility (m)	2,771.1 (2649.1–2893.2)	2,572.1 (2518.8–2625.5)	< 0.01	–	–
Distance to nearest water source (m)	805.1 (750.3–859.8)	824.5 (801.7–847.2)	0.49	–	–
Distance to nearest road (m)	645.0 (576.9–713.2)	475.0 (452.4–497.6)	< 0.001	1.03 (1.02–1.04)‡	< 0.001
Population density	947.5 (846.3–1048.7)	1,031.9 (985.2–1078.5)	0.17	–	–
Environmental					
Monthly mean rainfall (mm), prior month	199.5 (197.2–201.8)	204.5 (203.7–205.3)	< 0.001	0.94 (0.90–0.97)§	< 0.001
Monthly mean rainfall (mm), same month	198.6 (196.6–200.7)	202.3 (201.6–203.0)	< 0.001	–	–
Enhanced vegetation index, prior month	0.48 (0.48–0.48)	0.48 (0.48–0.48)	0.41	–	–
Enhanced vegetation index, same month	0.49 (0.49–0.50)	0.49 (0.49–0.50)	0.86	–	–
Tasseled cap wetness index, prior month	−0.25 (−0.25–−0.25)	−0.25 (−0.25–−0.25)	0.67	–	–
Tasseled cap wetness index, same month	−0.23 (−0.23–−0.23)	−0.23 (−0.23–−0.23)	0.13	–	–
Tasseled cap brightness index, prior month	0.57 (0.56–0.57)	0.57 (0.57–0.57)	0.97	–	–
Tasseled cap brightness index, same month	0.56 (0.55–0.56)	0.55 (0.55–0.55)	0.04	–	–
Day land surface temperature (°C), prior month	30.9 (30.7–31.0)	30.9 (30.8–30.9)	0.64	–	–
Day land surface temperature (°C), same month	30.2 (30.1–30.3)	30.2 (30.2–30.3)	0.22	–	–
Night land surface temperature (°C), prior month	20.8 (20.7–20.9)	21.0 (20.9–21.0)	< 0.001	–	–
Night land surface temperature (°C), same month	21.6 (21.5–21.7)	21.6 (21.6–21.7)	0.26	–	–
Diurnal difference land surface temperature (proxy for moisture), prior month	10.1 (10.0–10.2)	9.9 (9.9–10.0)	0.002	–	–
Diurnal difference land surface temperature (proxy for moisture), same month	8.6 (8.5–8.7)	8.6 (8.6–8.6)	0.73	–	–

OR = odds ratio; Ref = reference.

* Defined as seropositive to early transcribed membrane protein 5 antigen 1.

† Unweighted sample sizes.

‡ Per 100 m.

§ Per centimeter.

Nearly all risk factors were significantly associated with the risk of living in a cluster of elevated infection in the univariate analyses. However, in the final adjusted model, only household-level risk factors were maintained. Similar to the previous analyses, those living in households with increasing wealth had significantly less odds of living in a spatial infection cluster as compared with those living in the poorest wealth quintile. The odds of living in a spatial infection cluster increased for those living in households that owned livestock (aOR: 1.86; 95% CI: 1.25–2.77; *P* < 0.01), had rudimentary wall material (aOR: 2.22; 95% CI: 1.28–3.82; *P* < 0.01), had rudimentary floor material (aOR: 5.91; 95% CI: 1.68–20.7; *P* < 0.01), and were farther from the road (aOR: 1.08 per 100 m; 95% CI: 1.06–1.09; *P* < 0.001) (Table 5).

**Table 5:** Risk factors for living in a spatial case cluster in the study area, Artibonite, Haiti, *N* = 21,771*

Risk factor	Lives in spatial malaria cluster survey estimate, % or mean (95% CI) *N* = 782*	Does not live in spatial malaria cluster survey estimate, % or mean (95% CI) *N* = 20,989*	Univariate *P* value	Adjusted OR (95% CI)	Adjusted *P* value
Individual level					
Sex, female	51.7 (48.3–55.0)	55.0 (54.2–55.8)	0.06	1.08 (0.93–1.25)	0.34
Age category (years)			Overall 0.03		
0 to < 5	16.8 (13.9–20.1)	14.7 (14.1–15.3)	Ref	Ref	–
5 to < 15	27.3 (24.3–30.4)	24.7 (24.0–25.5)	0.81	1.11 (0.84–1.47)	0.45
15+	56.0 (52.4–59.0)	60.6 (59.7–61.4)	0.09	1.19 (0.93–1.51)	0.16
Febrile, yes	0.95 (0.43–2.09)	0.33 (0.25–0.45))	0.12	–	–
Did you sleep under a bed net last night? Yes	3.0 (1.6–5.8)	8.9 (8.1–9.8)	< 0.001	–	–
Live in same household during past 24 months? Yes	92.2 (88.2–94.9)	85.8 (84.7–86.8)	< 0.001	–	–
Recent travel within last 3 months? Yes	3.4 (2.2–5.2)	4.7 (4.3–5.2)	0.10	–	–
Household level					
Occupation of head of household (*N* = 18,579), agricultural/farmer	80.1 (70.8–87.0)	50.3 (48.4–52.2)	< 0.001	–	–
Head of household education level (*N* = 18,563), never attended	72.0 (62.7–79.6)	48.0 (46.1–50.0)	< 0.001	–	–
Caretaker education level (*N* = 9,890), never attended	77.1 (65.3–85.8)	49.1 (46.3–51.9)	< 0.001	–	–
Wealth index (quintiles)			Overall < 0.001		
Poorest	68.3 (59.6–75.9)	18.8 (17.8–19.8)	Ref	Ref	–
2nd	23.4 (16.7–31.8)	19.9 (18.7–21.2)	< 0.001	0.53 (0.33–0.86)	0.01
Middle	8.1 (4.5–14.3)	20.3 (18.9–21.9)	< 0.001	0.34 (0.16–0.72)	< 0.01
4th	0.2 (0.0002–0.01)	20.5 (19.0–22.1)	< 0.001	0.02 (< 0.01–0.11)	< 0.001
Wealthiest	–	20.5 (18.9–22.2)	< 0.001	1	–
Livestock ownership? Yes	81.0 (74.9–85.8)	69.0 (67.4–70.7)	< 0.001	1.86 (1.25–2.77)	< 0.01
Agricultural land ownership? Yes	72.6 (64.4–79.5)	68.2 (66.5–69.8)	0.27	–	–
Roof material					
Thatched, sod, mat, wood	10.4 (6.3–16.8)	2.6 (2.2–3.1)	< 0.01	–	–
Metal, cement, shingles	89.6 (83.2–93.7)	97.4 (96.9–97.8)			
Wall material					
None, bamboo, earth, mud, wood	82.8 (74.5–88.8)	39.4 (37.8–41.0)	< 0.001	2.22 (1.28–3.82)	< 0.01
Cement, stone, tile	17.2 (11.2–25.5)	60.6 (59.0–62.2)			
Floor material					
Earth, sand, wood	99.2 (97.4–99.8)	61.4 (59.6–63.1)	< 0.001	5.91 (1.68–20.7)	< 0.01
Cement, stone, tile	0.8 (0.2–2.6)	38.6 (36.9–40.4)			
Elevation (m)	568.9 (551.9–585.9)	267.8 (259.1–276.5)	< 0.001	–	–
Distance to nearest health facility (m)	4,271.3 (4031.9–4510.8)	2,539.2 (2486.2–2592.3)	< 0.001	–	–
Distance to nearest water source (m)	1,183.5 (1011.7–1355.4)	817.2 (794.7–839.8)	< 0.001	–	–
Distance to nearest road (m)	1,955.5 (1801.1–2110.0)	446.6 (424.5–468.7)	< 0.001	1.08 (1.06–1.09)†	< 0.001
Population density	273.4 (259.3–287.4)	1,036.7 (990.4–1083.0)	< 0.001	–	–
Environmental						
Monthly mean rainfall (mm), prior month	159.4 (153.4–165.4)	205.5 (204.7–206.2)	< 0.001	–	–
Monthly mean rainfall (mm), same month	168.7 (162.9–174.5)	203.1 (202.4–203.8)	< 0.001	–	–
Enhanced vegetation index, prior month	0.47 (0.47–0.48)	0.48 (0.48–0.48)	0.06	–	–
Enhanced vegetation index, same month	0.48 (0.47–0.49)	0.49 (0.49–0.50)	< 0.001	–	–
Tasseled cap wetness index, prior month	−0.26 (−0.26–−0.25)	−0.25 (−0.25–−0.25)	< 0.001	–	–
Tasseled cap wetness index, same month	−0.24 (−0.25–−0.24)	−0.23 (−0.23–−0.23)	< 0.001	–	–
Tasseled cap brightness index, prior month	0.57 (0.56–0.57)	0.57 (0.57–0.57)	0.32	–	–
Tasseled cap brightness index, same month	0.56 (0.56–0.56)	0.55 (0.55–0.55)	< 0.001	–	–
Day land surface temperature (°C), prior month	29.0 (29.0–29.1)	30.9 (30.9–31.0)	< 0.001	–	–
Day land surface temperature (°C), same month	28.4 (28.3–28.5)	30.3 (30.2–30.3)	< 0.001	–	–
Night land surface temperature (°C), prior month	19.4 (19.3–19.5)	21.0 (21.0–21.0)	< 0.001	–	–
Night land surface temperature (°C), same month	20.5 (20.4–20.6)	21.6 (21.6–21.7)	< 0.001	–	–
Diurnal difference land surface temperature (proxy for moisture), prior month	9.6 (9.5–9.8)	9.9 (9.9–10.0)	< 0.001	–	–
Diurnal difference land surface temperature (proxy for moisture), same month	7.9 (7.8–8.0)	8.6 (8.6–8.7)	< 0.001	–	–

OR = odds ratio; Ref = reference.

* Unweighted sample sizes.

† Per 100 m.

The trends in wealth and livestock ownership seen in the adjusted spatial infection cluster analysis were also shared in the adjusted spatial cluster of recent exposure analysis. Also, similar to the risk of recent exposure analysis, protective effects of elevation and monthly mean rainfall (prior month) were also seen. Additionally, lower population density decreased the likelihood of living in a spatial cluster of recent exposure (aOR: 0.94; 95% CI: 0.92–0.95; *P* < 0.001) (Table 6).

**Table 6:** Risk factors for living in a cluster of seropositivity to marker of recent exposure to P. falciparum* in the study area, Artibonite, Haiti, *N* = 21,102†

Risk factor	Lives in recent exposure cluster survey estimate, % or mean (95% CI) *N* = 1,576†	Does not live in recent exposure cluster survey estimate, % or mean (95% CI) *N* = 19,526†	Univariate *P* value	Adjusted OR (95% CI)	Adjusted *P* value
Individual level					
Sex, female	53.3 (50.7–55.9)	55.0 (54.2–55.9)	0.21	1.04 (0.92–1.17)	0.55
Age category (years)			Overall 0.80		
1 to < 5	14.0 (12.1–16.2)	14.7 (14.1–15.3)		Ref	–
5 to < 15	25.0 (22.7–27.4)	24.8 (24.1–25.6)		1.01 (0.81–1.26)	0.92
15+	61.0 (58.4–63.6)	60.5 (59.6–61.3)		1.03 (0.84–1.25)	0.78
Febrile, yes	0.56 (0.21–1.48)	0.33 (0.24–0.44)	0.42	–	–
Did you sleep under a bed net last night? Yes	9.6 (7.2–12.7)	8.7 (7.9–9.6)	0.40	–	–
Live in same household during past 24 months? Yes	86.0 (82.0–89.2)	85.9 (84.8–87.0)	0.98	–	–
Recent travel within last 3 months? Yes	6.5 (4.9–8.4)	4.6 (4.1–5.1)	0.04	–	–
Household level					
Occupation of head of household (*N* = 18,579), agricultural/farmer	43.8 (38.1–49.7)	51.5 (49.5–53.5)	0.03	–	–
Head of household education level (*N* = 18,563), never attended	46.4 (40.2–52.7)	48.7 (46.7–50.7)	0.49	–	–
Caretaker education level (*N* = 9,890), never attended	0.53 (0.45–0.62)	0.50 (0.47–0.53)	0.45	–	–
Wealth index (quintiles)			Overall < 0.001		
Poorest	23.9 (20.2–28.1)	19.5 (18.5–20.6)	Ref	Ref	Ref
2nd	12.2 (9.3–16.0)	20.6 (19.4–21.9)	< 0.001	0.68 (0.47–0.97)	0.04
Middle	11.5 (8.3–15.8)	20.6 (19.1–22.2)	< 0.001	0.90 (0.58–1.39)	0.63
4th	20.1 (15.5–25.6)	20.0 (18.5–21.7)	0.26	1.94 (1.24–3.02)	< 0.01
Wealthiest	32.3 (26.7–38.4)	19.2 (17.6–21.0)	0.07	3.91 (2.51–6.08)	< 0.001
Livestock ownership? Yes	59.0 (53.1–64.6)	70.2 (68.5–71.9)	< 0.001	0.51 (0.38–0.68)	< 0.001
Agricultural land ownership? Yes	67.1 (61.4–72.3)	68.4 (66.7–70.1)	0.65	–	–
Roof material					
Thatched, sod, mat, wood	5.7 (4.1–7.9)	2.6 (2.1–3.1)	0.001	–	–
Metal, cement, shingles	94.3 (92.1–95.9)	97.4 (96.9–97.9)			
Wall material					
None, bamboo, earth, mud, wood	42.4 (37.2–47.7)	40.2 (38.5–41.8)	0.46	–	–
Cement, stone, tile	57.6 (52.3–62.8)	59.8 (58.2–61.5)			
Floor material					
Earth, sand, wood	59.3 (53.5–65.0)	62.3 (60.5–64.1)	0.37	–	–
Cement, stone, tile	40.7 (35.1–46.5)	37.7 (35.9–39.5)			
Elevation (m)	235.5 (218.4–252.5)	278.1 (268.9–287.4)	< 0.001	0.73 (0.69–0.78)‡	< 0.001
Distance to nearest health facility (m)	2,697.7 (2515.4–2880.1)	2,573.6 (2518.5–2628.8)	0.28	–	–
Distance to nearest water source (m)	878.6 (797.5–959.7)	819.0 (795.5–842.5)	0.19	–	–
Distance to nearest road (m)	625.4 (533.2–717.7)	472.8 (449.4–496.3)	< 0.01	1.05 (1.04–1.07)	< 0.001
Population density	666.9 (616.4–717.4)	1,056.2 (1007.1–1105.4)	< 0.001	0.94 (0.92–0.95)‡	< 0.001
Environmental					
Monthly mean rainfall (mm), prior month	194.8 (191.3–198.3)	205.0 (204.2–205.8)	< 0.001	0.80 (0.75–0.84)§	< 0.001
Monthly mean rainfall (mm), same month	192.2 (189.4–195.0)	202.9 (202.2–203.6)	< 0.001	–	–
Enhanced vegetation index, prior month	0.51 (0.50–0.51)	0.48 (0.48–0.48)	< 0.001	–	–
Enhanced vegetation index, same month	0.53 (0.52–0.53)	0.49 (0.49–0.49)	< 0.001	–	–
Tasseled cap wetness index, prior month	−0.24 (−0.24–−0.24)	−0.25 (−0.25–−0.25)	< 0.001	–	–
Tasseled cap wetness index, same month	−0.22 (−0.22–−0.22)	−0.23 (−0.23–−0.23)	< 0.001	–	–
Tasseled cap brightness index, prior month	0.57 (0.56–0.57)	0.57 (0.57–0.57)	0.02	–	–
Tasseled cap brightness index, same month	0.55 (0.55–0.56)	0.55 (0.55–0.55)	0.10	–	–
Day land surface temperature (°C), prior month	31.4 (31.2–31.6)	30.8 (30.8–30.9)	< 0.001	–	–
Day land surface temperature (°C), same month	30.3 (30.2–30.4)	30.2 (30.2–30.3)	0.64	–	–
Night land surface temperature (°C), prior month	20.6 (20.5–20.7)	21.0 (20.9–21.0)	< 0.001	–	–
Night land surface temperature (°C), same month	21.1 (21.0–21.2)	21.7 (21.6–21.7)	< 0.001	–	–
Diurnal difference land surface temperature (proxy for moisture), prior month	10.8 (10.7–11.0)	9.9 (9.8–9.9)	< 0.001	–	–
Diurnal difference land surface temperature (proxy for moisture), same month	9.1 (9.0–9.3)	8.6 (8.5–8.6)	< 0.001	–	–

OR = odds ratio; Ref = reference.

* Defined as seropositive to early transcribed membrane protein 5 antigen 1.

† Unweighted sample sizes.

‡ Per 100 m.

§ Per cm.

## DISCUSSION

This large household survey was the first of its kind to characterize and systematically map malaria transmission foci in Haiti. Two predominant spatial case clusters (radii of 1.7 and 1.1 km with estimated populations of 478 and 267, respectively) in Verrettes were identified in similar locations for both infection status and marker of recent exposure to *P. falciparum*, suggesting recent and ongoing transmission in these areas, which corroborates results from another study conducted in this area.^30^ Two small (radii 0.20 and 0.07 km) unique spatial infection clusters in Verrettes were also identified. One larger (radius 2.69 km) unique cluster of recent exposure to *P. falciparum* was also detected in La Chapelle. The latter indicates an area with higher recent, but not current, malaria transmission. This is plausible because the incidence of malaria in La Chapelle in 2016 prior to the survey was higher than that of Verrettes (3.9 versus 2.1 per 1,000 persons, respectively) (MSPP, unpublished data).

The protective risk factors for malaria such as household wealth and individual bed net use identified in this study have been shown in other studies. As household wealth increases, risk for malaria tends to decrease, and this trend has been seen in Haiti and elsewhere, for both risk of malaria and risk of recent exposure to *P. falciparum*.^31^^–^^33^ Individual bed net use reported in the survey was low overall (5.9%); however, use of a bed net the previous night was still associated with reduced odds of malaria infection. This suggests that bed net use might be protective even in a setting with relatively low transmission and where the main vector of malaria transmission has exophilic biting tendencies that generally begin at sundown, such as in Haiti.^4^^–^^6^ In the Haitian context, LLIN acceptance is high when distributed free of charge.^34^

Interestingly, the crude elevations are higher among cases than controls; however, elevation is protective in the adjusted models, as has been shown in other studies. And although an increase in rainfall is generally considered a risk factor for malaria, the opposite was found in this study. This is unlikely due to the breeding, biting, or resting habits of *An. albimanus*, and more likely driven by cases located in the Terre Natte subcommune (western mountainous region of the study area), where 71 (44.1%) cases were found. At the time of the survey in 2017, there was no functioning health facility within 5 km of the subcommune boundaries. The health facility located in Terre Natte had not been functioning for several years prior to, during, and after the survey (PNCM, communication). Additionally, in Terre Natte, the monthly mean rainfall (prior month) among households was less (154 mm) than the remainder of the study area (202 mm).

Transmission of malaria in this high elevation of Haiti goes against the accepted niche of *An. albimanus*, the primary vector for *P. falciparum* in Haiti. Although the movement and introduction of malaria cases in the Terre Natte subcommune from lower-lying communities cannot be ruled out, autochthonous transmission with *An. albimanus* or other vectors must also be considered. Studies and a model have suggested that the range of *An. albimanus* may be increasing to higher elevations.^35^^,^^36^ Additionally, it is not clear whether the presence of other vectors such as *Anopheles pseudopunctipennis*, which have been described in Haiti and also been found at higher elevations, are also present at these highland or mountainous communities.^4^^,^^36^^–^^39^ Potentially, malaria introductions and autochthonous transmission may be acting in concert to foment outbreaks in these communities. However, the dynamics of this relationship, if it exists, has yet to be established.

Although numbers were low, not surprisingly, an individual who was febrile had an increased odds of current malaria infection as detected by RDT. Also, not surprisingly, this association was not found in the serology analyses, which modeled associations with recent exposure to malaria where any associated fever would have likely resolved.

Rudimentary wall material was also found to have significantly increased the odds of malaria than finished wall material. This has been found elsewhere, including in Haiti, and is likely due to rudimentary wall materials enabling mosquito entry more easily than finished wall materials.^33^^,^^40^ Living in a household with rudimentary floor material was also found to have significantly increased the odds of living in a spatial infection cluster. Further research into the effects of housing improvements on malaria in Haiti is warranted.

Sex and age categories were included in the models a priori. Sex did not have a significant effect in any models; however, increasing age categories had increased odds of both malaria and recent exposure. This is not uncommon because malaria occurs among individuals of all ages in areas of low transmission because immunities are not built or sustained with limited exposure.^41^

The household survey had several limitations. First, the duration was longer than expected and spanned the rainy and dry seasons (July 17 to October 4), with a pause in data collection from September 6 to 24 due to hurricane threats. Only one case and seven recent exposures were detected after the pause; however, 98% of the samples were already collected by this time. By design, to promote geographic variation in daily sample collection, up to 35 teams on any given day were deployed to different regions across the study area throughout the duration of the survey.

Limitations in the data include the potential for misclassification of recent travel among non-HoH or non-CT household members because recent travel was not asked directly of all household members by design for survey efficiency reasons. Instead, the HoH and CT were asked whether he or she traveled during the past 3 months, and if so, whether he or she had travel companions. If yes, the household member(s) who traveled during the (up to) five most recent trips was identified and the other household members were assumed to have not traveled. Therefore, any recent travel by a household member other than the HoH or CT, without the HoH or CT, was not captured. Additionally, because of the survey design, the HoH occupation and education levels of the HoH and CT were only suitable for univariate analyses so as not to drastically trim the sample size of the multivariable analyses. In each univariate analysis, individuals living in a household with an HoH who worked in an agricultural/farmer occupation had statistically significantly higher odds of the respective model outcome than those living in households with an HoH in other occupations.

This study demonstrated that individual, household, and environmental risk factors are associated with the odds of individual risk of malaria and recent exposure in La Chapelle and Verrettes, Artibonite Department, but that spatial clusters are primarily associated with risk factors at the household level. These findings can be incorporated into future algorithms or screening tools intended to identify high-transmission foci without the need for intensive household surveys. Additionally, this household survey identified two predominant spatial clusters of malaria that overlapped with clusters of recent exposure that led to further investigation and action by the Ministry of Health.

## Supplemental Material


Supplemental materials
