# Supplementary material for: Spatial Clustering and Risk Factors for Malaria Infections and Marker of Recent Exposure to Plasmodium falciparum from a Household Survey in Artibonite, Haiti
Source: Am J Trop Med Hyg. 2023 Jun 5;109(2):258–72. doi: 10.4269/ajtmh.22-0599 (PMC10397426; doi:10.4269/ajtmh.22-0599)
Supplement: Supplementary file 1 [file tpmd220599.SD1.pdf]

Supplementary Table 1. Reasons household survey was not completed due to unavailability.

**Reason for unavailability**	**N=1,465 n (%)**
The family or person is absent because they have traveled for business, vacation, or some other reason	805 (55.0)
The house is abandoned or locked	221 (15.1)
The family or person has moved to another house	191 (13.0)
The family or person is at the market	75 (5.1)
House under construction	5 (0.3)
Other reason*	168 (11.5)

*Other specify reason examples included the head of household had died since the census, the head of household owned two homes and was already surveyed, or the structure was not an inhabited, residential structure (e.g., a garden home, kitchen, spirit house where no one resides).

Supplementary Table 2. Individual reasons for blood draw refusal.

**Reason for blood draw refusal**	**N=831 n (%)**
Recently tested	226 (27.2)
Does not wish to participate/just refused/no other reason specified	182 (21.9)
Other medical condition	111 (13.4)
Fear	109 (13.1)
Belief	74 (8.9)
Child	64 (7.7)
Other reason*	49 (5.9)
Prefer health facility	11 (1.3)
Multiple reasons	5 (0.6)

*Other reasons examples included the head of household was not present and the individual was in a hurry.

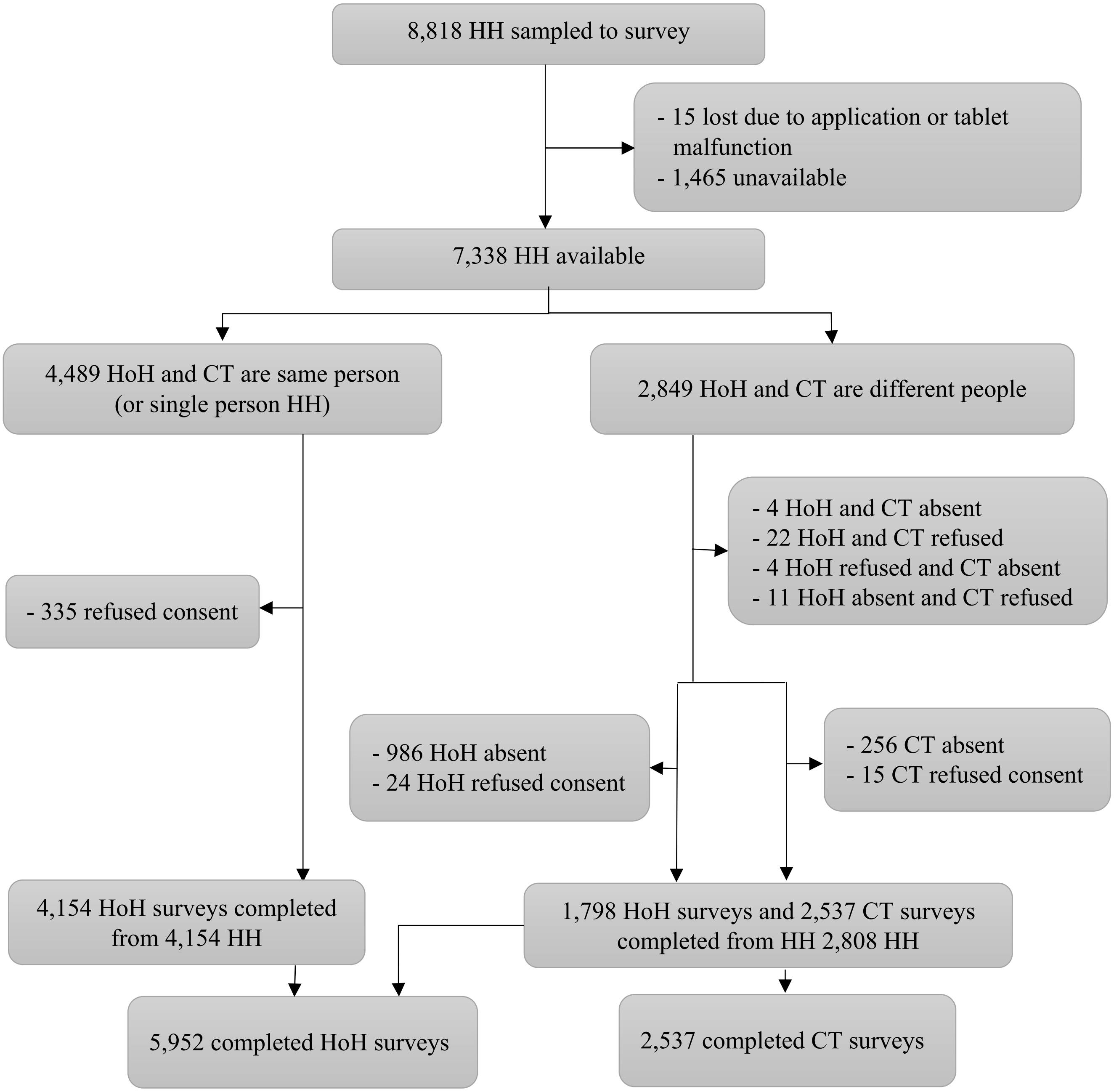

Supplementary Figure 1. Flow diagram illustrating the numbers of sampled households (HH), refusals, and completed head of household (HoH) and caretaker (CT) surveys among 6,962 HH with completed surveys, Artibonite, 2017.

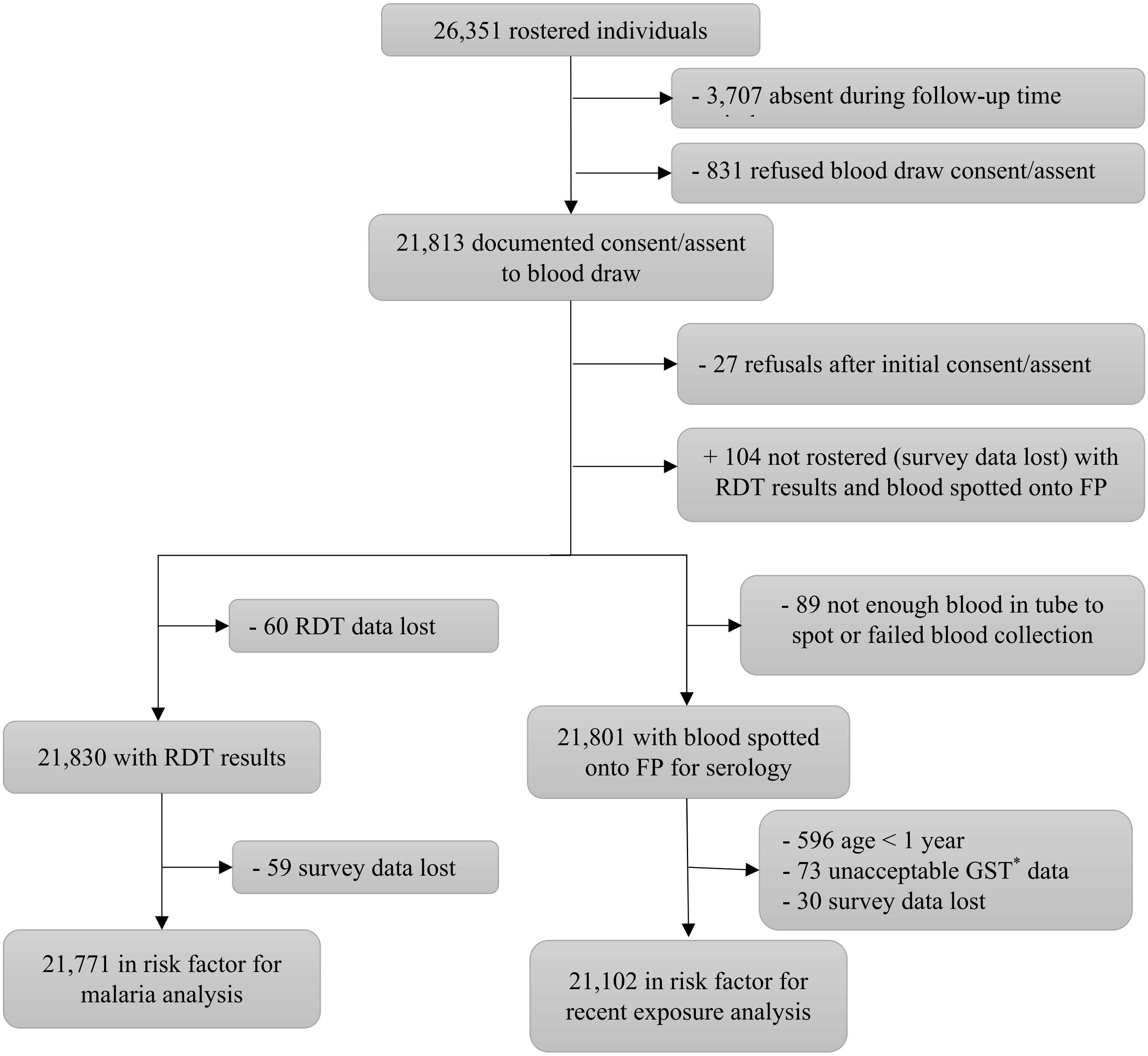

Supplementary Figure 2. Flow diagram of individuals, with rapid diagnostic test (RDT) results and blood spotted onto filter papers (FP) for serology testing included in the analyses, Artibonite, 2017. *GST = glutathione S-transferase
